# Supplementary material for: Analysis of PPARγ Signaling Activity in Psoriasis
Source: Int J Mol Sci. 2021 Aug 10;22(16):8603. doi: 10.3390/ijms22168603 (PMC8395241; doi:10.3390/ijms22168603)
Supplement: Supplementary file 1 [file ijms-22-08603-s001.zip › Supplemental materials_Analysis of PPARg signaling activity in psoriasis/Pathway models/Models images and html files/PPARG negative regulators and targets/1002917786.html]

MAP3K1 ---| PPARG


# Regulation MAP3K1 ---| PPARG

|  |  |
| --- | --- |
| URN | urn:agi-regulation:in-out:urn:agi-llid:4214:out:urn:agi-llid:5468:eff=negative |
| Connectivity | 2 |
| References | 1 |
| Effect | negative |

---
